# Trends in age at first sex in Uganda: evidence from Demographic and Health Survey data and longitudinal cohorts in Masaka and Rakai

**DOI:** 10.1136/sti.2008.034009

**Published:** 2009-03-13

**Authors:** E Slaymaker, J B Bwanika, I Kasamba, T Lutalo, D Maher, J Todd

**Affiliations:** 1 Centre for Population Studies, London School of Hygiene and Tropical Medicine, London, UK; 2 Rakai Health Sciences Program, Kalisizo, Rakai, Uganda; 3 MRC/UVRI Research Unit on AIDS, Entebbe, Uganda

## Abstract

**Objectives:**

To derive the best possible estimates of trends in age at first sex (AFS) among successive cohorts of Ugandan men and women based on all the data available from the Demographic and Health Surveys (DHS) and cohort studies in Masaka and Rakai districts.

**Methods:**

The datasets from the DHS, Masaka cohort and Rakai cohort were analysed separately. Survival analysis methods were used to estimate median AFS for men and women born in the 1950s–1980s and to compute hazard ratios for first sex, comparing later cohorts with earlier cohorts.

**Results:**

The DHS and Masaka data showed an increase in AFS in women in the more recent birth cohorts compared with those born before 1970, but this was less apparent in the Rakai data. Successive male cohorts in Masaka appeared first to have an increased AFS which subsequently decreased, a trend that was also apparent (but not significant) in the DHS data. Younger men in Rakai had an earlier AFS than those born before 1980.

**Conclusions:**

Women in Uganda who were born after 1970 have, on average, had sex at a later age than those born earlier. For men, AFS has not changed consistently over the period in question. Differences between Masaka and Rakai may reflect socioeconomic differences. Most of the change in AFS occurred too late to have contributed to the initial decline in the incidence of HIV.

Although not a strategy that can be maintained throughout a lifetime, avoidance of sexual intercourse is a reliable way to avoid contracting most sexually transmitted infections and of preventing unwanted conceptions. Abstinence has been a key component of many HIV prevention programmes in Africa and elsewhere.^1^^–^3 Abstinence usually means encouraging young people to delay their sexual debut with the intention of reducing their exposure to sex and sexual partners before committing to a stable and hopefully long-lasting partner. For HIV prevention, implicit in this message is that this permanent partner is uninfected, or at least less likely to be infected than other more casual partners.

There is considerable interest in measuring levels and trends in the key behaviours tackled by many HIV prevention programmes: abstinence, number of partners and condom use. Age at first sex (AFS) is a key indicator for measuring behaviour change in HIV prevention programmes.^1^ ^4^ ^5^ In many developing countries, AFS and age at first marriage (AFM) have occurred at older ages in recent years compared with 40–50 years ago.^6^ ^7^ The reasons for these changes are complex, but there are clear links between development and delayed AFS. It is also clear that behaviour change does not occur in isolation as risk behaviour is unlikely to change unless the social context is supportive of change. The timing of first sex is a product of agency and opportunity. Individuals may have limited autonomy to delay sexual debut if their first experience of sex is through coercive sex or rape.^8^^–^10

Uganda was the first country to report a generalised epidemic and to show a decline in the prevalence of HIV among the general population. Sexual behaviour data including AFS have been collected in several Demographic and Health Surveys (DHS) over the past 15 years,^11^ and have been collected from two population-based cohorts in south western Uganda. There is a considerable literature on changes in sexual behaviour and their influence on the decline in HIV prevalence in Uganda during the 1990s.^12^ ^13^ This highly charged debate has largely concluded that a multifactorial response—and not one simple behaviour change—was key to the decline in prevalence.^14^ ^15^ A secondary but crucial point is that data from the relevant time period are inadequate to settle the debate.^16^ ^17^

AFS is the only behavioural measure for which historical trends can be reconstructed using more recently reported recall data. These trends can be difficult to identify using data from a series of cross-sectional surveys because observed change could be due to differences in data quality between surveys or to changes in the composition of the population between the time of AFS and the time of the survey or between surveys. If these differences are related to AFS—for example, if those with a younger AFS tend to emigrate or to die prematurely—an artificial trend may be seen.

We determined the trends in AFS in Uganda by comparing repeated cross-sectional data from the DHS with data collected from two cohorts in rural Uganda. Changes in population structure do not affect the cohort data because information is collected on all individuals during the time they are resident in the area and retained for later analysis. The data therefore include anyone who subsequently died or moved away as well as immigrants to the area.

The quality of retrospectively reported data on AFS can be compromised by reporting bias due to accidental recall errors or through a desire to conform to health education messages. This can be assessed directly using cohort data and is discussed elsewhere in this issue. Although considerable variability in response is apparent, this does not have a directional effect on the results.^18^

## METHODS

Data were used from all the DHS conducted to date in Uganda (1988, 1995, 2000/1 and 2006) and from two longitudinal population-based studies, one in Masaka district and one in Rakai district. The DHS provides robust national estimates for different time points, and the methods by which the data are collected are openly available.^11^ The cohort data provide detailed information for two sets of respondents from two districts of Uganda, both of which have been extensively reported in publications over the past 15 years.^12^ ^19^^–^21

### DHS

Data were obtained for all the DHS conducted in Uganda. In 1988 only women were interviewed but in subsequent years (1995, 2000/1, 2006) data are available for men and women. The geographical range of the sample was restricted in 1988 compared with the later surveys. AFS, reported in whole years, is one of the first questions asked in the section on marriage and sexual behaviour and has been asked in the same way in all surveys. We have restricted our analysis to people who were born between 1950 and 1991 and had their first sex between 1960 and 2006; 973 respondents who were born before 1950 were excluded. We have used the unedited data from the DHS and have not corrected AFS for other reported ages in the DHS (age at first marriage and age at first birth). National rather than regional data were used because the number of respondents from the region that contains Masaka and Rakai districts was inadequate for this analysis.

### Masaka study

The Medical Research Council (MRC) general population cohort has been described previously.^12^ ^19^ Briefly, the study is located in a rural subcounty in Masaka district in south-western Uganda where an open cohort has participated in annual population-based surveys. In 1989 the census included 10 000 people residing in 15 villages, but in 1999/2000 this expanded to 18 000 residents in 25 villages. Survey staff administer a risk factor questionnaire and annually collect a blood sample for HIV testing. Starting in 1997, AFS was asked as the first question on sexual behaviour, in the same way in all surveys, and reported in whole years. For this paper, analysis was restricted to participants born between 1950 and 1994.

### Rakai study

The Rakai Health Sciences Program (RHSP) has, since 1994, conducted annual surveys using an open-community cohort of consenting persons aged 15–49 years resident in rural communities of Rakai District, south-western Uganda.^20^ ^21^ Surveys are conducted annually using a questionnaire that includes modules on both sociodemographic and behavioural characteristics. The same question on AFS was asked, in completed years, at the enrolment visit of a participant during study rounds 4 (1997–8) and 6–10 (1999–2004). This analysis was restricted to participants born between 1950 and 1989 and resident in 43 communities that have been followed since 1994.

### Statistical methods

For the two cohort studies, responses that were older than the age of the respondent at the time of the survey were excluded from the analysis. For each participant the most likely AFS was calculated as the age reported in 50% or more of the survey responses, or the mean age if no age was reported in 50% of the surveys. Stata Version 10 (Stata Corp, College Station, Texas, USA) was used for all analyses.

For all three studies we defined birth cohorts based on reported age in the DHS and reported date of birth in the cohort studies. Ten-year birth cohorts, using 1950–9 as the baseline cohort, were used for the analysis. Later analysis using 5-year birth cohorts was restricted to those born after 1 January 1970, whose first sex occurred when the prevalence of HIV was already high. The cohort born in 1970–4 was used as the baseline group for comparison.

For all data we smoothed the distribution by adding a random fraction of a year to each person’s reported AFS to account for the time between their birthday and the date of first sex. With this approach it is possible to estimate summary measures for the population in increments of <1 year. Ten replications of each of the datasets were created using different simulations of the random fraction and the Stata command *micombine* was used for the formal comparisons.^22^

Age-to-event analysis (ie, survival before sexual debut) was used to summarise the median age and interquartile range (IQR) of AFS. Curves plotting the cumulative proportion who have had sex at any given age are used to display the pattern of sexual debut.^23^ Cox regression models (accounting for the survey design for DHS data; Stata Version 10) were used to look for changes in the median AFS by birth cohort. For the DHS data, these analyses were repeated for each survey and by selected background characteristics.

## RESULTS

The number of male and female respondents in each of the DHS and from the Masaka and Rakai study by year of birth is shown in tables 1 and 2. These tables also show the median AFS by birth cohort for each data source. From the DHS, a consistent increase in the median AFS was seen for women and a similar trend was apparent for young men. However, the composition of the DHS changes over time as each survey captures a different set of birth cohorts.

**Table 1:** Number of male respondents by survey and year of birth, and median age at first sex (AFS) for each survey (for all DHS and for the Masaka and Rakai studies)

Year of birth	DHS					Masakacohort		Rakaicohort	
	1995	2001	2006	Total	Median AFS†	N	Median AFS	N	Median AFS
1950–9	384	260	205	849	18.1	492	18.5	852	18.1
1960–9	636	494	502	1632	18.2	843	18.2	2060	17.9
1970–9	767	631	690	2088	18.0	1389	18.0	4060	17.8
1980–9	25	524	874	1423	17.9	2781	18.5	2821	17.1
1990–4	0	0	232*	232*	18.1*	933	17.1	0	–
Total	1812	1909	2503	6224	18.0	6438	18.2	9793	17.7

DHS, Demographic and Health Survey.

*1990–1 only.

†Based on data from all surveys.

**Table 2:** Number of female respondents by survey and year of birth, and median age at first sex (AFS) for each survey (for all DHS and for the Masaka and Rakai studies)

Year of birth	DHS						Masakacohort		Rakaicohort	
	1988	1995	2001	2006	Total	Median AFS†	N	Median AFS	N	Median AFS
1950–9	1158	1171	777	360	3466	15.9	581	16.6	1039	15.9
1960–9	1948	2273	1604	1553	7378	16.1	1030	16.8	2196	16.0
1970–9	947	3199	2805	2497	9448	16.4	1768	17.0	5606	16.6
1980–9	0	131	2060	3366	5557	17.1	3235	17.5	4853	16.5
1990–94	0	0	0	755*	755*	–	829	16.9	0	–
Total	4053	6774	7246	8531	26604	16.4	7443	17.1	13694	16.4

DHS, Demographic and Health Survey.

*1990–1 only.

†Based on data from all surveys.

**Figure 1:**
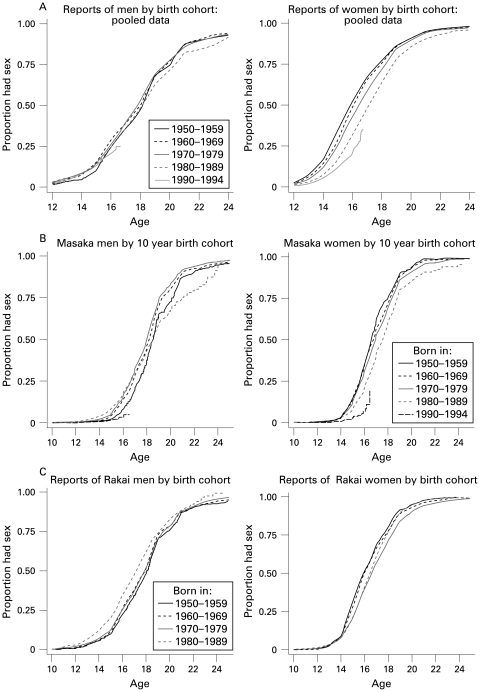
Survival curves (cumulative proportion that has had sex by age) for men and women by 10-year birth cohorts. (A) Pooled Demographic and Health Survey data; (B) Masaka study; (C) Rakai study.

Survival curves by 10-year birth cohorts are shown in fig 1. Figure 1A combines the data from all four of the DHS, and the curves suggest that there has been a change towards a later AFS for women. The pattern of the change appears to be different for men and women: the female cohorts born in the 1980s appear to be delaying first sex from the outset, whereas there has been little change in the proportion of men who have had sex at the younger ages. The survival curves for women in the Masaka study (fig 1B) and the Rakai study (fig 1C) show a similar pattern to those from the DHS, with the female cohorts of the 1970s, 1980s and 1990s showing a delay in AFS. In Masaka women the AFS is slightly higher than that reported in the DHS study and in the Rakai cohort (table 2 and fig 1), and the delay in AFS among the 1980s birth cohort appears to be smaller in the Rakai cohort than in the DHS data and the Masaka cohort. For men, there seems to be some delay in AFS in the 1970s and 1980s birth cohorts in Masaka, but an earlier AFS in the 1980s birth cohort in Rakai (fig 1).

Table 3 translates these survival curves into relative hazards for the 10-year birth cohorts taking a baseline of those born in 1950–9. There is strong evidence from the DHS and the Masaka study that women in the 1980s and 1990s cohorts are starting sex later, with significantly lower relative hazards for those born in the 1970s, 1980s and 1990s compared with the baseline cohort of the 1950s. For the women in Rakai a significantly lower hazard is seen for the cohort born in the 1970s, but there was no significant difference in hazard for starting sex between those born in the 1980s and those born in the 1950s. For men, both the DHS and the Masaka study showed a trend towards delayed AFS in the later birth cohorts, but the only significant effects were seen in Masaka with a higher relative hazard in those born in the 1970s and a lower hazard in those born in the 1990s. Conversely, the trend in Rakai was towards an earlier AFS, with a significantly higher relative hazard in the cohort born in the 1980s compared with those born in the 1950s.

**Table 3:** Crude hazard ratios (HRs) from Cox regression models showing the effect of 10-year birth cohorts on survival time to first sex (data from pooled DHS, Masaka study and Rakai study)

Year of birth	DHS		Masaka		Rakai	
	Crude HR	p Value	Crude HR	p Value	Crude HR	p Value
*Men*						
1950–9	1		1		1	
1960–9	1.08 (0.98 to 1.19)	0.130	1.19 (1.06 to 1.33)	0.004	1.03 (0.95 to 1.11)	0.5
1970–9	1.08 (0.99 to 1.18)	0.096	1.29 (1.15 to 1.44)	<0.001	1.05 (0.98 to 1.13)	0.16
1980–9	0.93 (0.83 to 1.05)	0.253	0.98 (0.87 to 1.11)	0.79	1.61 (1.49 to 1.74)	<0.001
1990–4	0.86 (0.61 to 1.21)	0.383	0.32 (0.19 to 0.64)	<0.001	–	–
Total	6113		5655		11211	
						
*Women*						
1950–9	1		1		1	
1960–9	0.94 (0.9 to 0.99)	0.017	0.92 (0.83 to 1.02)	0.11	0.96 (0.89 to 1.03)	0.3
1970–9	0.87 (0.83 to 0.92)	<0.001	0.83 (0.75 to 0.91)	<0.001	0.81 (0.76 to 0.87)	<0.001
1980–9	0.67 (0.63 to 0.71)	<0.001	0.62 (0.57 to 0.69)	<0.001	1.03 (0.96 to 1.11)	0.36
1990–4	0.37 (0.3 to 0.44)	<0.001	0.23 (0.15 to 0.35)	<0.001	–	–
Total	26531		6880		14808	

DHS, Demographic and Health Survey.

**Figure 2:**
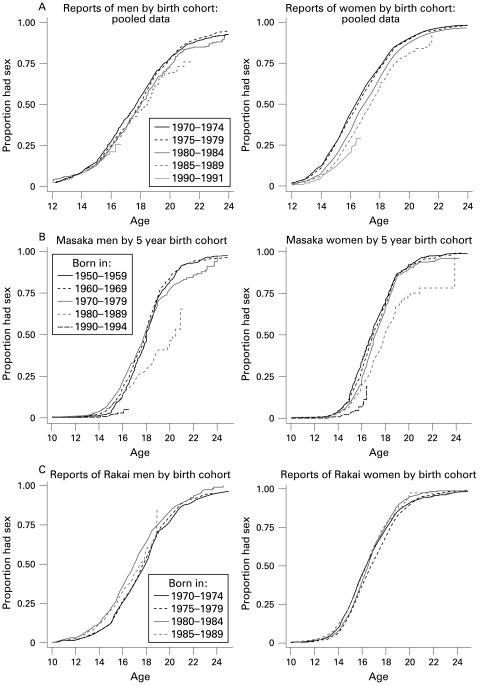
Survival curves (cumulative proportion who have had sex by age) for men and women born since 1970 by 5-year birth cohorts. (A) Pooled Demographic and Health Survey data; (B) Masaka study; (C) Rakai study.

In restricting the analysis to those born after 1 January 1970, the survival curves for 5-year birth cohorts are shown in fig 2 and the relative hazards in table 4. In the DHS and Masaka studies, men and women in the later cohorts (those born in 1980–4 and 1985–9) had delayed AFS (fig 2). The Rakai data show the opposite, with the later cohorts having earlier AFS. This pattern is clearly seen in the hazard ratios for AFS in table 4, which shows increased hazard in the later cohorts in Rakai but reduced hazards in the DHS and Masaka studies.

**Table 4:** Crude hazard ratios (HRs) from Cox regression models showing the effect of 5-year birth cohorts on survival time to first sex (data from pooled DHS, Masaka study and Rakai study)

Year of birth	DHS		Masaka		Rakai	
	Crude HR	p Value	Crude HR	p Value	Crude HR	p Value
*Men*						
1970–4	1		1		1	
1975–9	0.99 (0.89 to 1.1)	0.81	1.02 (0.91 to 1.15)	0.7	1.10 (1.03 to 1.17)	0.002
1980–4	0.88 (0.77 to 1.0)	0.059	0.94 (0.83 to 1.06)	0.29	1.59 (1.49 to 1.71)	<0.001
1985–9	0.82 (0.70 to 0.96)	0.011	0.51 (0.44 to 0.59)	<0.001	1.58 (1.42 to 1.76)	<0.001
1990–4	0.8 (0.57 to 1.12)	0.187	0.21 (0.12 to 0.34)	<0.001	–	–
Total	3662		4398		8013	
						
*Women*						
1970–4	1		1		1	
1975–9	0.97 (0.92 to 1.03)	0.301	0.93 (0.84 to 1.02)	0.13	0.94 (0.89 to 0.99)	0.016
1980–4	0.82 (0.77 to 0.86)	<0.001	0.84 (0.76 to 0.93)	0.001	1.20 (1.13 to 1.26)	<0.001
1985–9	0.67 (0.62 to 0.73)	<0.001	0.54 (0.48 to 0.61)	<0.001	1.37 (1.27 to 1.49)	<0.001
1990–4	0.44 (0.37 to 0.54)	<0.001	0.27 (0.18 to 0.41)	<0.001	–	–
Total	15734		5328		11707	

Restricted to those born on or after 1 January 1970.

DHS, Demographic and Health Survey.

Using data from the DHS, restricted to those born after 1 January 1970, comparisons can be made between those with different characteristics (table 5). AFS was delayed in later female birth cohorts, with a similar highly significant pattern in all regions (table 5). The delay in AFS was not seen in women with secondary education and was much smaller in women in the richest quintile. For men a smaller overall effect was seen (table 4). The delay in AFS in men born in the 1980s was seen as significant in urban areas and in those with no education (table 5).

**Table 5:** Hazard ratios (HRs) from Cox regression models showing the effect of 5-year birth cohorts on survival time to first sex (for pooled DHS data), stratified for area of residence, region, education and wealth

	Median AFS 1970–4 cohort	HRs by birth cohort				
		1970–4	1975–9	1980–4	1985–9	1990–1
*Men*						
All subjects (N)		1002	1041	831	558	230
All subjects (HR)	17.7	1	0.99	0.88	0.82*	0.8
Area (urban/rural)						
Urban	17.1	1	0.78*	0.76*	0.69*	0.48
Rural	17.9	1	1.04	0.91	0.85	0.88
Region						
Central†	17.5	1	0.95	0.99	0.96	0.64
Eastern	17.0	1	0.97	0.82	0.63*	0.43*
Northern	18.2	1	1.22	0.95	1.14	1.62*
Western	18.0	1	0.90	0.72*	0.67*	0.8
Education						
None	17.6	1	0.87	0.88	0.39	N/A
Primary	17.6	1	1.02	0.85	0.85	0.68
Secondary and above	18.1	1	0.97	0.97	0.84	1.71
Wealth ranking						
Poorest	17.3	1	1.0	0.86	0.73	1.3
Poor	18.4	1	1.36*	1.19	0.96	0.75
Middle	17.7	1	0.82	0.93	0.84	0.71
Rich	15.7	1	0.82	0.79	0.73	0.93
Richest	16.2	1	**0.93**	0.87	1.0	0.75
Year of survey						
1995	17.5	1	0.92	0.51	–	–
2001	18.1	1	1.02	0.95	0.6	–
2006	17.6	1	0.97	0.9	0.83*	0.85
						
*Women*						
All subjects (N)		4893	4532	3454	2101	754
All subjects (HR)	16.4	1	0.97	0.82***	0.67***	0.44***
Area (urban/rural)						
Urban	16.6	1	0.94	0.79***	0.67***	0.61**
Rural	16.3	1	0.99	0.84***	0.69***	0.42***
Region						
Central†	16.1	1	0.97	0.81***	0.61***	0.44***
Eastern	15.8	1	0.96	0.83***	0.68***	0.47***
Northern	16.5	1	0.97	0.82**	0.71***	0.34***
Western	17.0	1	0.96	0.82**	0.71***	0.49**
Education						
None	15.9	1	0.99	0.98	0.77	0.41
Primary	16.2	1	1.04	0.81***	0.7***	0.42***
Secondary and above	17.9	1	0.92	0.94	0.87	0.91
Wealth ranking						
Poorest	16.0	1	1.05	0.94	0.82	0.35
Poor	16.3	1	0.99	0.91	0.78**	0.36***
Middle	15.1	1	1.09	0.83**	0.6***	0.45**
Rich	15.0	1	0.9	0.7***	0.57***	0.43***
Richest	17.1	1	1	0.88	0.76**	0.75
Year of survey						
1995	16.3	1	0.98	0.67		
2001	16.6	1	1.04	0.89*	0.56***	–
2006	16.2	1	0.93	0.8***	0.65***	0.42***

Restricted to those born on or after 1 January 1970.

All birth cohorts compared with those born in 1970–4.

*p<0.05; **p<0.01; ***p<0.001.

†Central region contains Masaka and Rakai districts.

## DISCUSSION

Results from the DHS and from the Masaka study show a clear trend towards a delay in starting sex among women in Uganda, with those born in the 1980s reporting AFS about 1 year later than those born in the 1950s. A smaller delay was seen among women in Rakai who were born in the 1970s and the 1980s compared with those born in the 1950s. The change in AFS in men is less clear; in the DHS data no significant difference was seen between any of the male birth cohorts, and little evidence of a delay in AFS in either Masaka or Rakai. In the latest birth cohorts (born in the 1990s), the data from both DHS and Masaka indicate the possibility that AFS has been delayed even further in both women and men. However, this was based on small numbers and needs further follow-up to confirm the findings. These findings agree with other analyses of changes in sexual behaviour in young people in sub-Saharan Africa^6^ ^7^ and in Uganda.^13^ ^14^

By looking at the whole of the survival function rather than just the median AFS, it is clear that the pattern of change has been different for men and women and that the youngest women in the more recent cohorts are delaying sex but their male counterparts are not. In all graphs the women have sexual debut over a shorter period of time than the men, with many birth cohorts showing that some men are still virgin at the age of 25 years. This may show the choice that men have about sexual debut which may be denied to women, either through social pressure or through coercive sex.

The DHS findings show that the changes in the last 20 years have been greatest in those in the middle income quintiles and those with lower education (primary school or lower). This may be partly because more wealthy/highly educated young women have always been able to delay sexual debut, and it is only recently that the less wealthy and less educated have been able to access the same choices. The median AFS for women born in the 1970s who had secondary education and were in the richest quintile was 18.2 years compared with 16.3 years for all other women.

The timing of the observed changes in AFS relative to the decline in the prevalence of HIV in the early to mid 1990s (see fig S1 in the online supplement^24^^–^30) suggest that these changes have been a response to the danger of HIV infection, not a cause. To illustrate this, the survival functions can be plotted over calendar time. Based on DHS data, in 1980, 22% of 14–16-year-old women had had sex; in 1990 this had declined slightly to 20%, but had halved by 2000.

Changes in AFS may have helped to further reduce the incidence but cannot have been a cause of the initial decline. Young women need the knowledge and ability to delay first sex. Regardless of the contribution to HIV prevention, if women in Uganda are increasingly able to delay first sex, this would be a major achievement for the health messages that have been used over the last 20 years.

The changes in reported AFS in the Rakai study are different from those seen in the DHS and the Masaka study. There is some evidence that delaying AFS started earlier in Rakai, with women born in the 1970s who started sex in the late 1980s and early 1990s showing later AFS than those born in the 1950s. This could have occurred because the prevalence of HIV was higher at an earlier date and the preventive action by young women was initiated in an earlier birth cohort. However, the effect in the latest birth cohort of men in Rakai shows earlier AFS and does not fit with the explanation that changes in the sexual debut of young people are a response to the HIV infection seen in the community. The result of the changes in the most recent cohorts is a more similar experience for young men and women, around one-quarter of whom have started sex by about the age of 15 years in Rakai. Sex between partners of different ages can be a risk for HIV infection.^31^ ^32^ The availability of more sexually active young men may reduce age mixing in this community if young women increasingly choose partners close to their own age. This may be beneficial for HIV prevention, although any effect may be transient and dependent on other characteristics of the partnership networks.^33^ The difference seen between the Rakai and Masaka cohorts could be due to the inclusion of trading centres in the Rakai study which are not seen in Masaka, as these may provide some employment to younger single women and men, and keep them in the study area. Single men aged 15–24 years comprised 25% of the Masaka cohort and 29% of the Rakai cohort in 2006. Single women of the same age made up 21% of the Masaka cohort and just 18% of the Rakai cohort at the same time. The overall proportions of young single people and the sex ratios were therefore different between the cohorts.

There are several limitations to these analyses. We have combined data from repeated cross-sectional DHS and two cohort studies. There may be selection biases in the DHS, but these should not be present in the cohort studies.^23^ Different questionnaires were used for each data source. In the Rakai study, AFS was only asked at the enrolment into the cohort at round 4 and during rounds 6–10, so we have more information on some respondents. However, this is entirely at random as recruitment to the cohort is not related to first sex.

**Take-home messages**

- Ugandan women born since the 1970s have started sex later than their predecessors.
- Change in men’s age at first sex has not been directional.
- Changes in age at first sex are unlikely to have contributed to the initial decline in the prevalence of HIV in Uganda.

Others have shown that the quality of reporting of AFS can be variable and inconsistent, but this can be in either direction and should have no effect on the overall estimates.^18^ All data may be affected by social desirability bias as there have been extensive media campaigns in Uganda promoting safe sex and abstinence, but this should be similar across all sources. Although there have been health messages promoting abstinence and safe sex in Uganda, we did not see any difference in the responses of the same birth cohort over the four rounds of the DHS, which may indicate a limited bias due to social desirability. This effect may be more pronounced in the two cohort studies where the same people are seen every year and any messages would be reinforced over time, but there is no evidence for this in the Masaka data.^18^

A limitation of the cohort studies is the migration of young people from rural areas after completion of school. In Masaka, most young people (especially girls) do not remain at home, but either get married or migrate to larger towns or trading posts to search for work. This may skew the proportion of sexually active young women that remain in the cohort and may artificially raise the AFS if those who leave are never recruited into the cohort and have a younger AFS than cohort members.

All three studies cover a large population, so the effects we see have tight confidence limits and should give an accurate picture of any real changes in reported AFS in Uganda. The three studies indicate that large changes in AFS have been seen in women over the last 20 years. These changes may be influenced by the HIV epidemic seen in Uganda, but are unlikely to be the factor which explains the decline in HIV prevalence seen in Uganda in the 1990s. It is more likely that this is a positive side effect of the empowerment of women, especially young women, so that they can make their own choices about when and with whom they initiate sex.
